# Bone marrow mesenchymal stem cells increase motility of prostate cancer cells *via* production of stromal cell-derived factor-1*α*

**DOI:** 10.1111/jcmm.12010

**Published:** 2013-01-10

**Authors:** Barbara Mognetti, Giuseppe La Montagna, Maria Giulia Perrelli, Pasquale Pagliaro, Claudia Penna

**Affiliations:** Department of Clinical and Biological Science, University of TurinOrbassano, Italy

**Keywords:** Bone metastases, prostate cancer, mesenchymal stem cells, co-culture

## Abstract

Prostate cancer frequently metastasizes to the bone, and the interaction between cancer cells and bone microenvironment has proven to be crucial in the establishment of new metastases. Bone marrow mesenchymal stem cells (BM-MSCs) secrete various cytokines that can regulate the behaviour of neighbouring cell. However, little is known about the role of BM-MSCs in influencing the migration and the invasion of prostate cancer cells. We hypothesize that the stromal cell-derived factor-1*α* released by BM-MSCs may play a pivotal role in these processes. To study the interaction between factors secreted by BM-MSCs and prostate cancer cells we established an *in vitro* model of transwell co-culture of BM-MSCs and prostate cancer cells DU145. Using this model, we have shown that BM-MSCs produce soluble factors which increase the motility of prostate cancer cells DU145. Neutralization of stromal cell-derived factor-1*α* (SDF1α) *via* a blocking antibody significantly limits the chemoattractive effect of bone marrow MSCs. Moreover, soluble factors produced by BM-MSCs greatly activate prosurvival kinases, namely AKT and ERK 1/2. We provide further evidence that SDF1α is involved in the interaction between prostate cancer cells and BM-MSCs. Such interaction may play an important role in the migration and the invasion of prostate cancer cells within bone.

## Introduction

Prostate cancer is the second leading type of cancer in men in industrialized countries. Bone is a common site of metastasis also for prostate cancer, and these metastases represent the main cause of death for prostate cancer patients: approximately 70% of patients with prostate cancer have bone metastases at the time of death. The reason for the molecular and cellular predilection for prostate cancers to metastasize to bone is still the object of numerous studies. It is known that multiple factors, including the chemotactic responses to bone-derived factors and the interaction of prostate cancer cells with the bone microenvironment, are of paramount importance [1].

Bone marrow (BM) is the main source of multipotent mesenchymal stem cells (MSCs) that have been isolated and accurately characterized [2], together with the soluble factors they produce during *in vitro* culture [3]. Among these, a substantial role is played by the stromal cell-derived factor-1*α* (SDF1*α*, also known as CXC chemokine ligand 12, or CXCL12) [4], one of the most extensively studied chemokines endowed with numerous physiological functions, such as stem cell mobilization and homing [5].

More recently, SDF1α has gained further attention in the field of cancer biology [6]. SDF1α receptor, CXCR4, is essential for metastatic spread to organs where SDF1α is expressed. Therefore, it allows tumour cells to access cellular niches, such as the marrow, that favour tumour-cell survival and growth. Moreover, SDF1α can promote tumour angiogenesis by attracting endothelial cells to the tumour microenvironment and can stimulate survival and growth of neoplastic cells [7]. CXCR4 overexpression is known in more than 20 human tumour types, including ovarian [8], prostate [9], esophageal [10], melanoma [11], neuroblastoma [12]. Moreover, several lines of evidence suggest that the SDF1α/CXCR4 axis is involved in tumour progression and the development of distant metastases; this aspect has been highlighted particularly for breast carcinoma [13–15], which is characterized by the frequent appearance of bone metastasis. BM-MSCs are reported to interact with cancer cells in the tumour microenvironment and can be recruited from bone marrow to inflamed or damaged tissues by local endocrine signals. Many recent reports have pointed at tumour-stromal interactions as essential events for tumour progression [16, 17].

Clearly, MSCs promote tumour growth, invasion and angiogenesis [18–20], and are implicated in tumour formation of a cancer stem cell niche [21]. Moreover, they have been shown to promote growth and metastasis of colon cancer [22].

We questioned whether factors produced by BM-MSCs influence prostate cancer cells. In particular, we suggested that BM-MSCs production of soluble factors and SDF1α/CXCR4 interaction are crucial for attracting prostate tumour cells to the bone marrow niche. To verify this hypothesis, we examined the migration of the human prostate cancer cell line DU145 in an *in vitro* cell co-culture model with MSCs. We used transwell to study (i) how the medium released by MSCs can affect cell migration and (ii) the functional role of the SDF1α/CXCR4 interaction in this migration. Moreover, we verified whether soluble factors produced by MSCs can up-regulate kinases, namely ERK 1/2 and AKT, typically involved in SDF1α/CXCR4 interaction in other cell systems.

In this study, we demonstrate that BM-MSCs can attract prostate cancer cells, and that SDF1α is one of the molecules responsible of chemo-attraction. Our data confirm a role of SDF1α/CXCR4 in metastatic cascades of prostatic carcinomas and are consistent with an important role of MSCs in modifying cancer cells behaviour in the immediate cancer metastasis microenvironment.

## Materials and methods

### Materials

Reagents were purchased from Sigma-Aldrich (St. Louis, MO, USA) unless otherwise stated. Tissue culture plasticware was from Falcon (Franklin Lakes, NJ, USA).

### Cell culture

Human androgen independent DU145 prostate cancer cells were purchased from ATCC (Rockville, MD, USA).

Cells were maintained at 37°C in a humidified 5% CO_2_ atmosphere in RPMI 1640 containing 10 ml/l penicillin and streptomycin solution, NaHCO_3_ 2 g/l (7.5% w/v), 10% Foetal Bovine Serum (FBS).

### Bone marrow mesenchymal stem cells isolation and production of conditioned medium

Bone marrow cells were harvested from femurs of adult rats (body weight 450–550 g). The rats were housed in identical cages and were allowed access to water and a standard rodent diet *ad libitum*. The animals received care in accordance with Italian law (*DL-116*, *27 January 1992*), which complies with the Guide for the Care and Use of Laboratory Animals by the US National Research Council. The animals were anaesthetized with urethane (1 g/kg i.p.) and killed. Femurs were removed and cleaned from soft tissue. Marrow cells were obtained by inserting a 21-gauge needle into the upper end of the femur and flushing into the shaft 5 ml of complete α-modified Eagle's medium (αMEM) containing 20% FBS, 2 mM L-glutamine, 100 U/ml penicillin and 100 μg/ml streptomycin. Cell suspensions (10 ml of final volume from each rat) were dripped from the lower end of both femurs into a 50-ml sterile tube containing 40 ml of complete medium. The cells were then filtered through a 70 μm nylon filter (Falcon) and plated into one 75-cm^2^ flask. They were grown in complete αMEM containing 10% FBS, 2 mM L-glutamine, 100 U/ml penicillin and 100 μg /ml streptomycin at 37°C and 5% CO_2_ for 3 days. The medium was then replaced with fresh medium and the adherent cells were grown to 90% confluence to obtain samples defined here as mesenchymal stem cells (MSCs) at passage zero (P0). The P0 MSCs were washed with PBS and detached by incubation with 0.25% trypsin and 0.1% EDTA for 5–10 min. at 37°C. Complete medium was added to inactivate the trypsin. The cells were centrifuged at 450 r.p.m. for 10 min., resuspended in 10 ml complete medium, counted manually in duplicate using a Bürker's chamber and plated as P1 on 58-cm^2^ plates at densities of 2000 cells/cm^2^. Complete medium was replaced every 3–4 days over the 18–24-day period of culture.

We previously demonstrated that BM-MSCs isolated with this procedure in our laboratories are CD90 positive and CD34/CD45 negative and that under opportune stimuli they can differentiate into adipocytes, muscle and osteoblast [23–25]. The BM-MSCs were therefore included in this study and used accordingly to the protocols described below.

Conditioned medium was collected after 3 day of culture, centrifuged at 4000 r.p.m. for 5 min. at 4°C to eliminate cells and cellular debris, and freshly used for migration assays or cell culture, or frozen.

### Western blotting

Cells were seeded in 10 cm-diameter Petri dishes, cultured as described until sub-confluence, when medium was replaced with conditioned medium or with fresh αMEM, both additioned with 2% FBS. Following 8 hrs incubation, the medium was removed and the cell monolayer was first washed with PBS, then covered with ice-cold PBS and incubated for 5 min. to facilitate detachment. Subsequently, adherent cells were gently scraped (TPP scraper, Trasadingen, CH), collected and centrifuged at 2500 r.p.m. for 5 min. at 4°C. The pellet was then resuspended in 50 μl of RIPA buffer (Sodium chloride 150 mM, 1% Triton X-100, 0.5% Sodium deoxycholate, 0.1% SDS, Tris 50 mM with addition of 10 μl/ml protease inhibitor, 1.54 mM Sodium orthovanadate and 10 mM Sodium fluoride), placed in ice for 1 hr and gently shuffled every 20 min. to facilitate the membrane breakup. The mixture was then centrifuged at 13,200 r.p.m. for 30 min. at 4°C, the supernatant collected and protein content quantified with the Bradford assay (1 μl RIPA suspension/999 μl Bradford solution 1:5, Bio-Rad, Hercules, CA, USA): samples were read with a spectrophotometer (Beckman DU® 640 Spectrophotometer, Brea, CA, USA) at 595 nm wavelength. Fifty-eighty micro grams of proteins were resolved in the Invitrogen system (Carlsbad, CA, USA) by SDS-PAGE gels at 10% of polyacrylamide SDS gels in denaturing conditions, then transferred onto polyvinylidene difluoride membranes (GE Healthcare, Buckinghamshire, UK), and immunoblotted according to Penna *et al*. [26].

Blots were probed with primary polyclonal antibody suspended in TBS Tween 0.1%: anti-AKT (developed in mouse, 1:800), anti-pAKT (Ser473) (developed in rabbit, 1:500), anti-ERK1/2 (developed in mouse, 1:800), anti-pERK1/2 (developed in mouse, 1:500), anti-Vinculin (developed in rabbit, Sigma-Aldrich 1:1000). Vinculin was used as an internal control.

Secondary antibodies were suspended in TBS Tween 0.025% and used at the following concentrations: HRP- conjugated anti-Mouse 1:6000 (Amersham-GE Healtcare, Buckinghamshire, UK) and anti-Rabbit 1:8000 (Santa Cruz Biotechnology, Santa Cruz, CA, USA).

After first and secondary antibody incubation, the membranes were treated with chemiluminescent substrate and enhancer (Immun-Star™ HRP Chemioluminescent Kit – Bio-Rad, Hercules, CA, USA), followed by exposure to X-ray film (Kodak BioMax light film, Sigma-Aldrich) and finally developed and fixed in Kodak GBX developer and Kodak GBX fixer respectively.

The molecular weight ladder PageRuler™ Plus Prestained Protein Ladder *(*Fermentas, Vilnius, Lithuania) was used in each experiment.

Bands were quantified using the ImageJ software.

Phosphorylation levels of AKT and ERK were expressed as ratio pAKT/AKT and pERK/ERK. All data were expressed as modification relative to baseline (control conditions).

### Assessment of cell morphology

DU-145 morphology was considered after exposure to MSC conditioned medium for 6 hrs.

Cells were grown in complete medium with 10% FBS in adequate chambers mounted on plastic microscope slides (Lab-Tek Chamber Slide w/cover – swell – Permanox slide sterile – NY, USA – Nunc™); in each chamber 2000 cells/200 μl medium were seeded and allowed to adhere overnight. The following morning, the culture medium was replaced with MSC conditioned medium or with an adequate control medium; at the end of incubation cells were fixed in glutaraldehyde 2%, allowed to dry and stained in crystal violet 0.1%.

Each field was photographed under optical microscope (Leica DC 100) at 100× magnification using the BRESSER® MikroCam 3 Mpx camera.

### 3D migration assay

The transwell migration assay was used to measure the three-dimensional movement of the cells as described in Gambarotta *et al*. [27]. Migration assays were performed in transwells (BD Falcon™ cell culture inserts incorporating polyethylene terephthalate – PET – track-etched membranes with 8.0 μm pores at the density of 6 ± 2 × 10^4^/cm^2^).

Cells (10^5^) resuspended in 200 μl of culture medium containing 2% FBS were seeded in the upper chamber of a Transwell (cell culture insert, no. 353097, BD Biosciences, Franklin Lakes, NJ, USA) on a porous transparent polyethylene terephthalate membrane (8.0-μm pore size, 1 × 10^5^ pores/cm^2^). The lower chamber (a 12-well plate) was filled with culture medium containing 2% FBS or with conditioned medium containing 2% FBS.

When migration test was performed in the presence of SDF1α, 20 ng/ml [28] of this factor (Immunotools, Friesoythe, Germany) were added to the medium in the lower chamber. When migration test was performed in presence of a specific inhibitor of CXCR4, cells were preincubated for 30 min at 37°C gently shaking with 100 nM AMD3100 [29] (Sigma-Aldrich) before seeding in the transwell.

The 12-well plates containing cell culture inserts were incubated at 37°C in a 5% CO_2_ atmosphere saturated with H_2_O.

After 6 hrs of incubation, cells attached to the upper side of the membrane were mechanically removed using a cotton-tipped applicator. Cells that migrated to the lower side of the membrane were rinsed with PBS Ca/Mg (Na_2_HPO_4_ 8 mM, NaCl 0.14 M, CaCl_2_•2H_2_O 1 mM, MgCl_2_•6H_2_O 1 mM, KCl 2.7 mM, KH_2_PO_4_ 1.5 mM), fixed with 2% glutaraldehyde in PBS for 20 min. at room temperature, washed five times with water, stained with 0.1% crystal violet and 20% methanol for 20 min. at room temperature, washed five times with water, air-dried and photographed using the BRESSER® MikroCam 3 Mpx camera, with an optical microscope (Leica DC 100) at 100× magnification. Five pictures were randomly chosen per well, and used to count the migrated cells with ImageJ software using cell-counter plug-in. Results from different experiments (performed at least three times in duplicate) were expressed as mean ± SD.

### Statistics

Statistical analyses were performed by one-way or two-way anova, and *P* < 0.05 was considered significant. If not differently specified, data are expressed as the mean percentage ± SD percentage referred to control as baseline.

## Results

### DU-145 migration and morphology

**Fig. 1:**
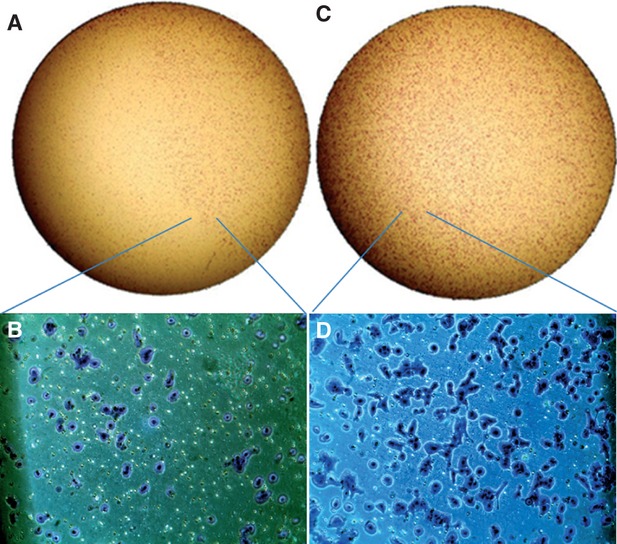
DU145 underwent migration test in basal conditions (control medium) or under chemotactic stimulus represented by medium conditioned by 3 days culture of bone marrow-mesenchymal stem cells. Complete and detailed transwell view: control medium (**A** and **B**) and MSC conditioned media (**C** and **D**). (**A** and **C**, stereoscopic microscope at 40× magnification; **B** and **D**, light microscope at 100× magnification).

**Fig. 2:**
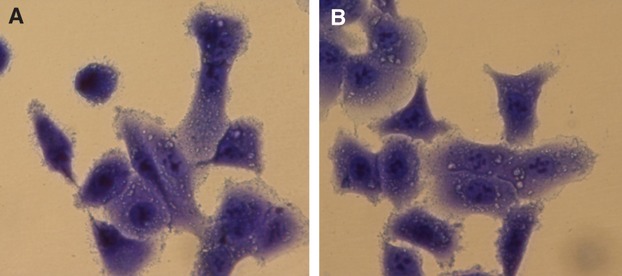
Morphological analysis of DU145 exposed for 6 hrs to control medium (**A**) or to MSC- conditioned medium ((**B**), optical microscope at 400× magnification). One representative image.

Conditioned medium significantly increased the rate of migration of DU145 (+76.42% ± 4.37, *P* = 0.006) compared with control (Fig. 1). On the other hand, DU-145 did not show evident morphological changes when exposed for 6 hrs to conditioned medium (Fig. 2).

### AKT and ERK activation

**Fig. 3:**
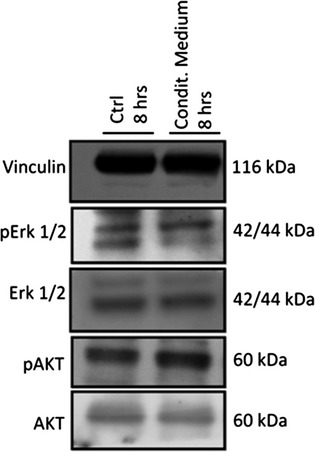
Activation of AKT and ERK following 8 hrs culture in control (first lane) or conditioned medium (second lane). Vinculin as internal control.

Eight hours incubation in MSC conditioned medium provoked in DU-145 an increase in pAKT/AKT ratio of 71.2% ± 6.4 *versus* control (*P* = 0.014; Fig. 3).

pERK/ERK ratio increased of 38.6%, compared with control.

### Influence of SDf1α in DU145 migration

**Fig. 4:**
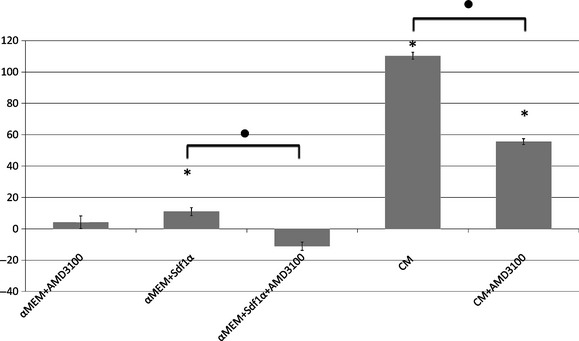
DU145 migration in control (αMEM) or conditioned (CM) medium, with or without SDF1α and CXCR4 blocker AMD3100. *significantly different from control conditions, considered as baseline. ^•^significantly different from each other (each significance reported in graph has *P* < 0.05).

Addition of SDF1α to control medium significantly stimulated DU145 migration, whereas blocking its receptor with AMD3100 significantly inhibited migration (Fig. 4).

Addition of AMD3100 greatly decreased the attractive effect of conditioned medium (Fig. 4); this result suggests the presence of SDF1α in MSC conditioned medium and its important role for migration in these conditions.

## Discussion

With a transwell co-culture system [30], we demonstrate that BM-MSCs produce soluble factors, including SDF1α, which can influence the behaviour of prostate cancer cells, namely their motility and their intracellular prosurvival kinases. These factors induce pro-survival kinase activation and may contribute to the homing and survival of cancer cells within the bone.

Metastasis is regulated by several signalling pathways in the cancer cells as well as in the microenvironment. Prostate cancers preferentially metastasize to the skeleton, and considerable research effort has been devoted to understanding the unique interaction between prostate cancer epithelial cells and the bone microenvironment. Human prostate cancer metastases home within the haematopoietic stem cell niche and colocalize with haematopoietic stem cells in the bone marrow [31].

Among the factors produced *in vitro* by BM-MSC, a good candidate in promoting migration is SDF1α, whose role in cancer biology has widely been described, to such an extent that it also gained a place of paramount importance in clinical settings [6, 32, 33].

DU145 express SDF1α receptor CXCR4 [32, 34] whose role in promoting cellular migration and invasion *in vitro* has already been tested [35]. In different cell types, activated CXCR4 exerts its biological effect [7, 36] initiating the downstream protein kinase B (AKT)/mitogen-activated protein kinases (MAPK) signalling pathway, leading to alteration of gene expression, actin polymerization, cell skeleton rearrangement and cell migration. These data are in line with our findings in prostate cancer cells. In fact, when DU145 were grown in the presence of BM-MSC conditioned medium for 8 hrs, AKT and ERK phosphorylation rates increased significantly.

Moreover, while supplementation of standard culture medium with SDF1α significantly increases cell migration, adding AMD3100-CXCR4-specific blocker to BM-MSC conditioned medium decreases cell migration. By blocking such SDF1α -CXCR4 axis, through AMD3100, we have shown that this axis covers a pivotal role in prostate cancer cell migration and that it is probably crucial in MSC conditioned medium attractive effect.

Nevertheless, SDF1α probably is not the only factor responsible for the cell migration in these conditions, because despite blocking its effect, cells still have a migration rate 55% higher than control.

Methodological considerations: We have cultured a human cell line in a conditioned medium obtained from MSC belonging to a different species, but this system has already been validated [30] as several authors have already done [37–39]. Furthermore, the homology degree is high between human and rat SDF1α. A rapid ‘BLAST Protein’ alignment pointed out high identity value (92%), no gaps and a very significant E-value (5e^−43^). Crystallography and functionality studies showed that the most important SDF1α portion is represented by two amino acids responsible for activating its receptor CXCR4: Lys-1 and Pro-2 [40]. No difference in any region of interest responsible for binding CXCR4 exists between human and rat protein. About the only substitution present (^65^Asp → ^65^Ser), it is not reported as possible vitiating bond with the receptor.

*In conclusion*, our work underlines the importance of factors produced by BM-MSCs in modifying the invasive behaviour of prostate cancer cells. We provide elements that one of these factors is SDF1α. These data therefore further support the exploitation of the SDF1α/CXCR4 axis as a therapeutic target for prostate cancer.
